# The Cognitive-Vestibular Compensation Hypothesis: How Cognitive Impairments Might Be the Cost of Coping With Compensation

**DOI:** 10.3389/fnhum.2021.732974

**Published:** 2021-10-01

**Authors:** Emilie Lacroix, Naïma Deggouj, Martin Gareth Edwards, Jeroen Van Cutsem, Martine Van Puyvelde, Nathalie Pattyn

**Affiliations:** ^1^VIPER Research Unit, LIFE Department, Royal Military Academy, Brussels, Belgium; ^2^Institute for Research in Psychological Science (IPSY), Université Catholique de Louvain, Louvain-la-Neuve, Belgium; ^3^Institute of Neuroscience (IONS), Université Catholique de Louvain, Louvain-la-Neuve, Belgium; ^4^Otorhinolaryngology Department, Cliniques Universitaires Saint-Luc, Brussels, Belgium; ^5^Human Physiology and Sports Physiotherapy Research Group, Vrije Universiteit Brussel, Brussels, Belgium; ^6^Brain Body and Cognition Research Group, Department of Psychology and Educational Sciences, Vrije Universiteit Brussel, Brussels, Belgium; ^7^Clinical and Lifespan Psychology, Department of Psychology and Educational Sciences, Vrije Universiteit Brussel, Brussels, Belgium

**Keywords:** vestibular, cognitive effort, cost, compensation, effort

## Abstract

Previous research in vestibular cognition has clearly demonstrated a link between the vestibular system and several cognitive and emotional functions. However, the most coherent results supporting this link come from rodent models and healthy human participants artificial stimulation models. Human research with vestibular-damaged patients shows much more variability in the observed results, mostly because of the heterogeneity of vestibular loss (VL), and the interindividual differences in the natural vestibular compensation process. The link between the physiological consequences of VL (such as postural difficulties), and specific cognitive or emotional dysfunction is not clear yet. We suggest that a neuropsychological model, based on Kahneman’s Capacity Model of Attention, could contribute to the understanding of the vestibular compensation process, and partially explain the variability of results observed in vestibular-damaged patients. Several findings in the literature support the idea of a limited quantity of cognitive resources that can be allocated to cognitive tasks during the compensation stages. This basic mechanism of attentional limitations may lead to different compensation profiles in patients, with or without cognitive dysfunction, depending on the compensation stage. We suggest several objective and subjective measures to evaluate this cognitive-vestibular compensation hypothesis.

## Introduction

Animal models and artificial stimulation studies on healthy human participants have delivered a growing body of evidence supporting a clear link between the vestibular system, emotional and cognitive impairments. This body of research consistently shows that postural imbalance which appears after (artificially created) vestibular damage is linked to cognitive changes, mostly related to space perception difficulties (Zheng et al., 2006; Lenggenhager et al., 2008; Lopez et al., 2010; Machado et al., 2012a,b; Ferrè et al., 2013a,b; van Elk and Blanke, 2014; Besnard et al., 2015; Deroualle et al., 2015). However, according to the clinical experience from Ear, Nose and Throat doctors (ENT) and their multidisciplinary teams, a high variety of patient profiles do not match this scientific evidence. For example, patients might present with a vestibular pathology associated with mild residual postural instability, but show no objective cognitive impairment measured by neuropsychological tests. At the same time, patients frequently complain of subjective emotional or cognitive difficulties, some variables missing in animal or healthy human artificial stimulation studies. Disentangling these different dimensions could help to disentangle the complex variety of observed patients profiles. Too few investigations have tried to quantify the specific contribution of each potential variable, and the results of the literature exploring vestibular-damaged patients profiles remain heterogeneous.

We present a novel hypothesis to explore the heterogeneous clinical profile of vestibular-damaged patients. Taking into account their degree of postural imbalance; their objective cognitive neuropsychological performances; and their degree of subjective cognitive, physical, and emotional complaints would lead to a comprehensive approach. We focus on the potential role of cognitive effort that patients have to invest to compensate their vestibular pathology. To quantify this effect, we apply a neuropsychological model, based on Kahneman’s Capacity Model of Attention, which will allow to integrate the existing findings from the literature in the framework of our hypothesis. The contribution of this model to clinical observations of a variety of patients’ profiles will be discussed, as well as a protocol that could be applied retrospectively on (un)published data. We are convinced that this cost-benefits approach could shed new light on clinical vestibular research.

### Animal and Artificial Stimulation Research

In rodent animal studies, vestibular loss (VL) is typically associated with spatial memory and navigation impairments (Russell et al., 2003; Baek et al., 2010; Besnard et al., 2012; Machado et al., 2012a); as well as with increased anxiety-like behaviour (Machado et al., 2012b). Moreover, spatial memory impairments seem to persist in time, at least up to 14 months after bilateral vestibular deafferentation (BVD), suggesting that cognitive deficits may be permanent despite a possible adaptation to the physical symptoms such as oscillopsia (Baek et al., 2010).

Similarly, studies on healthy human participants have also demonstrated specific cognitive impairments using artificial vestibular stimulation. For example, galvanic vestibular stimulation (GVS) modulated spatial perception bias in a bisection line task (Ferrè et al., 2013a) and random number generation (Ferrè et al., 2013c). Caloric vestibular stimulation (CVS) changed the perception of body part position in space, relative to body schema causing a bias in perceived object size, hand length, and hand width (Lopez et al., 2012). Vestibular stimulation caused by a rotatory chair influenced self-centred mental imagery, but not 3D object mental rotations (Deroualle et al., 2015).

These observations in animal and healthy human artificial stimulation research have led to patient studies, which have attempted to replicate results and identify common neural pathways. However, the generalisation to patient studies is complex for several reasons. Firstly, animal studies mostly use maze tasks for practical reasons, creating a literature bias toward the investigation of spatial memory compared to other cognitive functions. This under-representation of other cognitive functions makes the comparison with clinical population more difficult, as patients report many other cognitive decrements. Secondly, the understanding of the mechanisms of how artificial vestibular stimulation influences cognition is not yet well understood (Grabherr et al., 2015). Finally, the acute temporary character of the stimulation influence on cognition may not be an appropriate comparison to long-term chronic vestibular pathology.

Regarding patient studies, the variety of cognitive and emotional measures impairs the comparison to studies using artificial stimulation. Original patient studies have mostly used subjective questionnaires, consistently showing significant increases of emotional, physical, and cognitive complaints compared to control participant responses (Eagger et al., 1992; Yardley et al., 1992; Yardley and Putman, 1992; Godemann et al., 2004; Gómez-Alvarez and Jáuregui-Renaud, 2011; Alghwiri et al., 2013; Lahmann et al., 2015; Lacroix et al., 2016; Semenov et al., 2016; Liu et al., 2019). Comparison with animal and artificial stimulation research is difficult, as no questionnaires are used in animal research and very few questionnaires have been used with human artificial stimulation research. Fortunately, recent patient studies have included more objective neuropsychological measures (such as virtual mazes, computerised reaction time tasks, etc…), allowing some comparison.

### Variability of Results in Vestibular-Damaged Patient Studies

Objective neuropsychological assessment through computerised measures has contributed to a better understanding of VL patient cognition. Specific cognitive deficits have been identified for spatial cognition, short-term memory, executive functions, processing speed, and visuospatial abilities, particularly in patients with bilateral vestibular loss (BVL) when compared to patients with unilateral vestibular loss (UVL) or healthy controls (Grabherr et al., 2011; Popp et al., 2017; Deroualle et al., 2019). However, contrary to the global coherence observed in animal and stimulation studies, patient studies show less straightforward results. Several additional reasons can be identified for this discrepancy, mostly highlighting methodological differences between protocols.

Similarly to animal studies, some vestibular-damaged patient research has used orientation tasks such as the Virtual Morris Water Task (VMWT). Chronic BVL patients demonstrate impairments in this task, which are associated with a decreased hippocampal volume (Schautzer et al., 2003; Brandt et al., 2005). However, this structural change has not always been found in other studies of BVL (Cutfield et al., 2014) or UVL patients (Hüfner et al., 2007). In addition, studies exploring body perception in space have demonstrated depersonalisation symptoms (where one feels detached from one’s own body) in BVL and UVL patients (Sang et al., 2006; Jáuregui-Renaud et al., 2008a,b), but a subsequent study failed to evidence these effects using a subjective questionnaire in chronic BVL patients (Deroualle et al., 2017). So far, it remains unclear whether VL patients present specific cognitive deficits, such as space or numerical processing; or if the effects are rather more general cognitive deficits involving executive functioning (Risey and Briner, 1990; Moser et al., 2017). Whereas the extent of VL can be controlled in animal studies through surgical or chemical procedures, patient research needs specific physiological measures to assess the degree of this VL. Caloric testing (Mean Caloric Response – MCR, values from caloric irrigation in°/s from both ears with cold −30°C- and warm −44°C- water), vestibular evoked myogenic potentials (VEMP; registering information from two muscles effectors and allowing testing of otolithic receptors) or the video head impulse test (VHIT; measuring high acceleration for the six canals) provide complementary information about the current statute of the VL. However, it remains unclear whether and how these physiological measures are related to cognitive deficits.

Popp et al. (2017) evidenced a correlation between the degree of VL measured trough the MCR and two tasks measuring visuospatial abilities and memory in BVL patients. They also found a correlation between the VHIT outcomes and some aspects of memory and executive functions; for both UVL and BVL patients. However, no correlations were found between those physiological measures and processing speed, nor with the Corsi Block Tapping task. Those inconsistencies underline the complexity of establishing mechanistic links between the different dimensions. Other studies have searched for a link between physiological measures of the VL, cognitive and emotional impairments, and brain changes. For example, Helmchen et al. (2009) showed that UVL patients who recovered the best after the VL (at least at the physiological level, measured by the MCR), had a higher increase in grey matter volume (GMV; inferior insular temporal GMV increase), as a sign of their recovery. At the same time, their results demonstrated a volume increase in the vestibular insular cortex and superior temporal gyrus (STG) that was negatively correlated with the patient’s subjective vestibular disability score (SVDS), indicating that patients with higher subjective clinical complaints had a higher increase in these cerebral areas. On the other hand, no correlation was found in a subsequent study (Göttlich et al., 2016) between hippocampus volume and patient’s subjective measures [Vertigo Handicap Questionnaire (VHQ) (Tschan et al., 2010), Vertigo Symptom Scale (VSS) (Tschan et al., 2008), and SVDS]; nor with the quantitative assessment of the vestibulo-ocular reflex (VOR gain).

In addition, vestibular pathologies frequently accompany sensorineural hearing loss (SNHL) and many patient studies have not adjusted for this comorbidity (Smith, 2021). Recent research showed that cognitive function could be affected differently by each pathology, with specific challenges in immediate memory and language tasks for SNHL patients, and worse performance in attention tasks for VL patients (Dobbels et al., 2019). Statistically significant differences on the VMWT have been found between VL patients and healthy controls in studies where some VL patients (only one or two patients on the total sample) had mild hearing loss (Brandt et al., 2005; Kremmyda et al., 2016); whereas no differences were found in a larger VL patients sample when adjusting for hearing status (Dobbels et al., 2020).

Although the results presented above could be linked to the different measures used, the variety of vestibular pathologies studied could also provide a potential explanation. The many different types of vestibular pathologies (Ménière’s disease, vestibular neuritis, vestibular schwannoma, vestibular migraines, Benign Paroxysmal Positional Vertigo, vestibular nerve resection, or vestibular areflexia), as well as the different types of recovery a patient can experience, add complexity to this research field and warrant further investigation.

### Variety of Pathophysiology and Clinical Expression of the Patient Recovery

The early stage of a vestibular dysfunction is associated with diminished postural and oculomotor control, abnormal body perception in space, and autonomic symptoms. Fortunately for patients, VL triggers a vestibular pathway reorganisation called vestibular compensation, allowing for rapid improvements in postural control, action control, and improved body perception in the environment (Lacour et al., 2016). This vestibular compensation mechanism has been described as composed of three stages: Restoration, Habituation, and Adaptation. In addition, within the adaptation stage, a distinction is made between “sensory substitution” and “behavioural substitution.” Sensory substitution means that patients rely (intentionally or not) on other sensory modalities such as visual or somesthetic information in order to compensate for the impaired vestibular input. Behavioural substitution indicates that patients use other neural networks to mimic or replace vestibular function (Lacour et al., 2016).

Although compensation mechanisms are increasingly documented in animal research, there is a level of idiosyncrasy in human patient recovery that cannot be fully explained by the animal models. While some patients very rapidly succeed in returning to a normal balance; others only partially recover at the postural level, with a highly variable functional impact on their quality of life. The type of VL (UVL versus BVL) may partially explain the variety of compensation profiles in patients (Lacour et al., 2009). However, even in similar pathology, such as unilateral vestibular deafferentation patients (UVD), at least 20% of the patients may present persistent complaints of postural imbalance and incomplete long-term compensation (Reid et al., 1996; Halmagyi et al., 2010). Functionally compensated patients regarding the physiological impairment may nonetheless continue to present subjective complaints about their quality of life, with emotional and cognitive difficulties. These dimensions can be measured with specific questionnaires (Lacroix et al., 2016). Although several premorbid patient characteristics such as age (Gauchard et al., 2012); psychological factors (Yardley and Redfern, 2001); illness perception and coping strategies (Ribeyre et al., 2016); or the level of physical activity (Gauchard et al., 2013) seem to play a role in the recovery process, the way these different variables interact remains largely unknown.

### The Complexity of Cognitive-Vestibular Compensation Assessment in Patients

Compensation mechanisms of VL patients are typically investigated with various physiological measures, among which the improvement of the gain and phase of the VOR trough saccades (VOR) (Curthoys, 2000; Curthoys and Halmagyi, 2007; Macdougall and Curthoys, 2012; Ranjbaran et al., 2016); the postural score changes on dynamic posturography platforms (Gauchard et al., 2012; Parietti-winkler et al., 2016); or changes in the GMV (Helmchen et al., 2011; Hong et al., 2014). However, these assessments focus solely on the vestibular compensation at the physiological level. Furthermore, it is not always possible to implement these measures in clinical settings, with a varying degree of access to diagnostic resources (Agrawal et al., 2020). Most of the time, patient recovery is evaluated based on the clinical reduction of physical symptoms, such as a better postural control. Therefore, the persistence of subjective emotional or cognitive complaints such as agoraphobia, persistent fatigue or attentional disorders usually leads to supplementary (neuro)psychological consultations, where standard gold-standard measurements are not always sensitive enough to detect specific impairments.

Whereas it is widely accepted that postural recovery can vary from one patient to another, little is known about the associated subjective emotional or cognitive impairments, which might be the cost of a successful postural recovery. Guidetti et al. (2008) compared 50 unilateral labyrinthine-defective patients (without vertigo) to healthy controls using the Symptom Check List questionnaire (SCL-90; Derogatis et al., 1976) and several objective cognitive measures. These authors report that patients showed significantly higher levels of subjective anxiety and lower scores on the objective visual memory Corsi block task. However, no correlation analyses were performed between the measures, and no physiological compensation measures such as postural control were recorded. This type of analysis is essential if we want to understand whether patients presenting subjective physical or emotional complaints (despite postural compensation), also present specific cognitive neuropsychological impairments.

## Determining the Cognitive and Emotional Cost to Maintain a Functional Postural Balance After a Vestibular Damage

### Allocation of Resources Models

Various theoretical models attempt to explain how individuals allocate (willingly or not) resources when facing challenging actions, and what could be the cost of this allocation in terms of fatigue (Pattyn et al., 2018). In sleep research, it is well established that sleep loss and fatigue decrease the individual resources available to the task and increases the effort required to perform the task (Williamson et al., 2011). This compensatory model postulates that fatigue will primarily affect the secondary task activities, since primary task activities are protected (Robert and Hockey, 1997). In the vestibular domain, dual-task paradigms (where a participant performs a postural and a cognitive task at the same time) are similarly used to demonstrates competition between the cognitive resources needed to complete two tasks and a resulting cost to performance (Bigelow and Agrawal, 2015). Therefore, we advocate to apply a neuropsychological model to take into account the degree of perceived subjective compensation (at the physical, emotional, and cognitive level) in addition to objective physiological and cognitive measures.

### The Kahneman’s Capacity Model of Attention Adapted to Vestibular Damaged Patients

According to Kahneman’s Capacity Model of Attention (Egeth and Kahneman, 1975), there is a limited quantity of cognitive resources that can be allocated to any given task. Therefore, Kahneman’s model applied to VL predicts that a patient with successful physiological compensation (where cognitive resources are successfully used to maintain postural control, thereby preventing falls), would have reduced cognitive resources for other cognitive tasks in comparison to patients with non-successful compensation (i.e., where no cognitive resources are used for vestibular compensation) (Bigelow and Agrawal, 2015). Unlike sleep loss, where the compensatory efforts have to be provided temporarily in specific situations (when the restoration of sleep can be achieved later), the loss of vestibular information requires a continuous adaptation of the body to maintain a proper balance. Therefore, it is highly plausible that the cost of adaptation will affect specific cognitive abilities. This cost might fluctuate and increase with time, depending on the compensation stage, and consequently affect patients’ emotions and quality of life.

**FIGURE 1:**
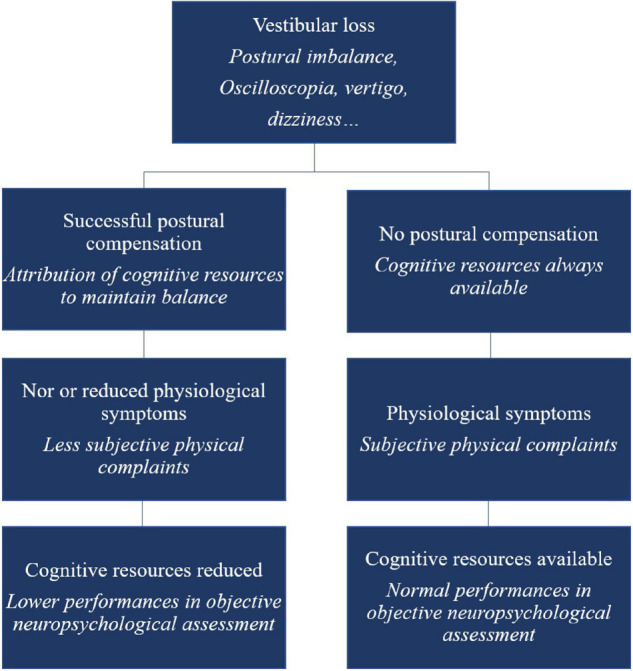
Schematic overview of the cognitive-vestibular compensation hypothesis – adaptation of the Kahneman’s Capacity Model of Attention to vestibular-damaged patients.

This hypothesis is in contrast to the traditional view, which assumes that the more physiologically or physically affected vestibular-damaged patients would show more cognitive or emotional disorders. We suggest that non-compensated patients (no use of cognition to compensate for posture) would not show difficulties in cognition as all their cognitive resources remain available. On the contrary, after successful physiological compensation, the loss of resources (dedicated to maintain balance) will affect cognitive abilities. With postural recovery achieved, patients’ subjective perception of their physical capacities could be positively affected, leading to a counter-intuitive observation: less complaining patients (at least at the physical level) would be more cognitively impacted (see Figure 1). This cognitive-vestibular compensation hypothesis could explain an apparent absence of group effect in some studies (as the different types of compensation could cancel each other out amongst the different patients depending on their compensation stage).

### Evidence Supporting the Cognitive-Vestibular Compensation Hypothesis

Although our hypothesis has never been investigated as such, there is tentative evidence supporting the assumption that only vestibular-damaged patients with successful compensation would show specific cognitive impairments. Redfern et al. (2004) evaluated the cognitive profiles of 15 UVL patients that were described as “well-compensated” (showing no symptoms of dizziness or postural deficits). When compared to controls in a dual-task paradigm, they demonstrated significantly slower reaction times during a choice and inhibitory reaction time task (the secondary task) while performing a postural task (the main task). However, three of the patients were described as “not perfectly well-compensated” (with abnormal results in posturography or vestibulo-ocular function) and these three patients showed faster reaction times than the other patients. It is possible that the three patients were only in the early stage of their compensation adaptation process and had sufficient cognitive resources available for the cognitive tasks. These preliminary results suggest that different compensation profiles may interfere with cognitive abilities. However, until now, the relationship between cognitive impairments and the various degrees of postural compensation has not been systematically investigated, and more research is needed to explore our hypothesis.

## Future Perspectives

Future research investigating how cognitive impairments might be the cost of coping with compensation would benefit from a degree of standardisation in assessment, including subjective and objective measures.

Regarding subjective assessment, we suggest using specific questionnaires to systematically determine how patients describe their own level of compensation. One validated gold standard questionnaire, the Dizziness Handicap Inventory (DHI; Jacobson and Newman, 1990), provides specific subscales evaluating physical, functional, and emotional self-perception of the patient’s vestibular state. It has been demonstrated that the functional subscale is related, at least partially, to GMV increase in visual and cerebellar areas; and therefore may be used as a potential sign of vestibular compensation (Helmchen et al., 2009; Hong et al., 2014; Lacour et al., 2016). Future studies could use the DHI to separate patients into subgroups, based on the scores for these subscales. Using a large patient group, it should be possible to analyse retrospectively if patients with higher versus lower levels of physical complaints show different objective cognitive results.

Regarding our cognitive-vestibular hypothesis, we predict that patients with higher levels of physical complaints are less physiologically compensated, and therefore will show preserved cognitive abilities. We propose to use specific subjective cognitive measures to test this hypothesis. The cognitive-failure questionnaire (CFQ; Broadbent et al., 1982), or the neuropsychological vertigo inventory (NVI; Lacroix et al., 2016), may offer helpful insight into patients’ own perception of their cognitive state. These questionnaires have already demonstrated their sensitivity by allowing for the identification of different profiles among different types of VL (Liu et al., 2019). However, it has not been possible so far to determine whether patients with higher levels of subjective cognitive complaints have higher objective cognitive deficits and what would be their state of physiological compensation. To the best of our knowledge, this has never been measured in such a holistic approach.

Regarding objective cognitive assessment, we thus propose that future research should include challenging assessments, taking into account the degree of cognitive effort required by the tasks. A recent study testing vestibular-damaged children yielded a distinction between dynamic (involving a “mental movement” during the execution to solve the task, such as in mental rotation) and static tasks [not involving mental movement, such as when performing a target detection task (Lacroix et al., 2020)]. We suggest that the cognitive tasks involving dynamic processes require greater cognitive resources than the static ones, and therefore are more likely to be sensitive to successful vestibular compensation. Alternatively, the level of cognitive resources involved in the tasks could also be estimated based on the amount of executive functions involved in the tasks. Executive functions have been shown to play a role in gait disturbances, and several simple tasks to assess these can easily be used in clinics (Yogev-Seligmann et al., 2008).

Finally, at the physiological level, we propose to investigate the role of compensatory ocular saccades as it seems that different patterns of saccadic response may predict different profiles of patient compensation (Macdougall and Curthoys, 2012). Correlations between objective and subjective cognitive measures on the one hand, and ocular saccades on the other hand, would allow to understand the seemingly random inter-individual differences in patients populations. In addition, physiological measures of brain volume and brain connectivity modifications could also help define different compensatory profiles. Neuroanatomical studies have previously demonstrated that CVS measuring the vestibular impairment were correlated with structural brain changes such as GMV (Helmchen et al., 2011; Hong et al., 2014; Lacour et al., 2016). Recent research also shows asymmetric cerebellar hyperactivity in patients with vestibular migraine, which could be linked to compensation after vestibular rehabilitation (Liu et al., 2020). Based on these findings, we suggest that patients who are compensated at the postural level (hence with mild clinical signs of vestibular impairment and lower subjective complaints) could exhibit an increase in GMV in specific areas such as visual cortices and cerebellum, similar to what has been observed by Hong et al. (2014). We suggest that this increase could be linked to changes in performance in the objective neuropsychological measures, differentiating between compensated and non-compensated patients. Reciprocally, the increases in GMV that Hong et al. (2014) found in the vermis and the prefrontal cortex could be related to visual dependence.

If our cognitive-vestibular compensation hypothesis is incorrect, and deafferentation is the sole cause of cognitive difficulties, VL patients with reduced objective cognitive performance should present a high level of physical and subjective cognitive disorders, whatever their degree of physiological compensation. Measuring compensation in vestibular-damaged patient is challenging, and interesting new perspectives have recently emerged such as trying to harmonise physiological measurements through a compensation index based on functional balance performance (Verbecque et al., 2021). The use of a new computational model of the vestibular system may also contribute to more fine-grained measures of cognitive costs associated with postural compensation (Mast and Ellis, 2015; Ellis and Mast, 2017). Cognitive rehabilitation such as mental imagery training in BVL patients could reduce physiological symptoms particularly in BVL patients that learn to rely more on anticipated sensory input and less on the impaired sensory measures (Ellis et al., 2018).

Investigating the cognitive-vestibular compensation hypothesis would allow for a better understanding of how the compensation mechanism operates; whether the patient is aware of this adaptation process; and which measures can be used to disentangle between compensated and non-compensated profiles. It would also open the door to the publication of non-significant results of objective cognitive function deficits in vestibular-damaged patients, when these data contrast with significant subjective cognitive or physiological measures. The exact cognitive cost of vestibular compensation might thus be objectivated.

## Data Availability Statement

The original contributions presented in the study are included in the article/supplementary material, further inquiries can be directed to the corresponding author/s.

## Author Contributions

EL wrote the manuscript. ME and ND critically revised the manuscript for important intellectual content. ND supported the initial funding. JV and MV made significant contributions to the manuscript. NP critically revised the manuscript and coordinated the collaboration to write the manuscript and provide the second funding. All authors contributed to the article and approved the submitted version.

## Conflict of Interest

The authors declare that the research was conducted in the absence of any commercial or financial relationships that could be construed as a potential conflict of interest.

## Publisher’s Note

All claims expressed in this article are solely those of the authors and do not necessarily represent those of their affiliated organizations, or those of the publisher, the editors and the reviewers. Any product that may be evaluated in this article, or claim that may be made by its manufacturer, is not guaranteed or endorsed by the publisher.

## Abbreviations

BVL: bilateral vestibular loss
CVS: caloric vestibular stimulation
DHI: dizziness handicap inventory
GVS: galvanic vestibular stimulation
MCR: mean caloric response
NVI: neuropsychological vertigo inventory
SVDS: subjective vestibular disability score
UVD: unilateral vestibular deafferentation
UVL: unilateral vestibular loss
VEMP: vestibular evoked myogenic potentials
VHIT: video head impulse test
VHQ: Vertigo Handicap Questionnaire
VL: vestibular loss
VOR: vestibulo-ocular reflex
VSS: Vertigo Symptom Scale.
